# Prevalence of depressive symptoms in adolescents living with HIV in Johannesburg, South Africa

**DOI:** 10.4102/sajpsychiatry.v30i0.2208

**Published:** 2024-11-12

**Authors:** Vuyiswa Gantsho, Mvuyiso Talatala, Nokuthula Mdaka

**Affiliations:** 1 Department of Psychiatry, Faculty of Health Sciences, University of the Witwatersrand, Johannesburg, South Africa; 2 Department of Psychiatry, Chris Hani Baragwanath Academic Hospital, Johannesburg, South Africa; 3 Department of Psychiatry, West Rand Health District, Krugersdorp, South Africa

**Keywords:** depressive symptoms, adolescents, HIV, PHQ-A, South Africa

## Abstract

**Background:**

Adolescents living with HIV (ALWHIV) are more vulnerable to developing depressive symptoms. Despite this knowledge, the screening for depression is not routinely integrated into HIV treatment programmes.

**Aim:**

The study aimed to determine the prevalence of depressive symptoms among ALWHIV.

**Setting:**

The study was carried out in the primary healthcare clinics and an antiretroviral clinic situated in a district hospital, West Rand District, Johannesburg.

**Methods:**

This was a cross-sectional study of 125 ALWHIV. The modified Patient Health Questionnaire for Adolescents (PHQ-A) was used to screen for depressive symptoms with a score of ≥ 5 deemed significant. A distress protocol was used for immediate psychological intervention.

**Results:**

The prevalence of depressive symptoms was 44.8% and the majority of ALWHIV reported mild-moderate symptoms (36.8%). Overall, 25.6% of adolescents had suicidal behaviours. Those with psychosocial difficulties and those who reported a previous suicide attempt were 3.6 (adjusted odds ratio [aOR] 3.59, 95% confidence interval [CI]:1.17–11.03) and 6.9 (aOR 6.93, 95% CI:1.39–34.55) times likely to develop depressive symptoms, respectively.

**Conclusion:**

A high prevalence of depressive symptoms was found in ALWHIV. Psychosocial difficulties and previous suicide attempts were predictive factors for depressive symptoms. This highlights the unmet need for the integration of mental health screening and overall mental health services into adolescent HIV programmes.

**Contribution:**

This study emphasises an urgent need for routine mental health screening and prompt psychosocial support in ALWHIV.

## Introduction

It is estimated that over 2 million adolescents between the ages of 10 and 19 years are living with either perinatally or horizontally acquired human immunodeficiency virus (HIV) globally, and 90% of these adolescents live in sub-Saharan Africa.^1,2,3^ Despite achievements in HIV prevention strategies, South Africa has the highest rate of HIV in the world with a prevalence of 6.5% among the older adolescent group between the ages of 15 and 19 years and 2.4% in children younger than 15 years.^2,4^ This prevalence is higher in females.^4^ Depression, suicidal behaviours and anxiety are the most common mental health conditions that co-occur with HIV in adolescents.^4,5,6^ Despite this high co-morbidity and previous recommendations, the integration of mental healthcare in HIV treatment programmes still lags behind in Africa and other poorly resourced settings.^5^

There is evidence supporting that adolescents with HIV are particularly more vulnerable to developing depressive symptoms compared to the general population.^7,8^ This is linked to the bidirectional relationship of HIV and depression with one exacerbating the other.^7,8^ Being infected with HIV is a severe psychological stressor that predisposes to depression.^7,8^ The presence of depression in adolescents living with HIV (ALWHIV) accelerates the progression of HIV and is associated with poorer clinical outcomes of HIV such as poor adherence, the presence of opportunistic infection, poor viral suppression, steeper declines in cluster of differentiation 4 (CD4) counts and more rapid progression to acquired immunodeficiency syndrome (AIDS).^7,8^

The prevalence of depressive symptoms among ALWHIV ranges from 4.4% to 59% across studies.^7,8,9,10,11,12,13,14,15^ In a Ugandan study, ALWHIV were screened for depressive symptoms using the Center for Epidemiological Studies Depression Scale (CES-D) and 46% of participants reported depressive symptoms.^8^ The prevalence of depression among 562 ALWHIV was 18.9% in a cross-sectional study conducted by Kim et al. in Malawi using a validated Chichewa version of the Beck Depression Inventory version-II (BDI-II) and the Children’s Depression Rating Scale-Revised (CDRS-R).^7^ South African studies of ALWHIV, although few, have reported prevalence rates of depression among adolescents similar to other countries. In a cross-sectional study by West et al., 8% of ALWHIV attending a primary healthcare (PHC) clinic in Johannesburg screened positive for depressive symptoms using the Children’s Depression Inventory Short Form (CDI), which is a self-reported tool.^10^ In a survey of 1053 ALWHIV from Eastern Cape province, South Africa, 46% of participants screened positive for depressive symptoms using CDI.^16^ The variation in the prevalence of depression in all these studies may be related to the differences in the screening tools that have been used by the different researchers as well as the different population profiles and sites where the research was conducted. In addition, as the screening tools are not diagnostic tools, further assessments would need to be conducted to confirm the clinical diagnosis of depression according to the Diagnostic and Statistical Manual of Mental Disorders (DSM-5).

Several factors associated with higher prevalence rates of depressive symptoms in ALWHIV have been described in the literature. These factors can be categorized into sociodemographic factors, psychological factors and clinical HIV-related factors. The sociodemographic factors described include being female, having a poor socioeconomic status, AIDS-related orphanhood and having an unemployed caregiver.^7,17,18^ In previous literature, AIDS-related orphans were found to have a higher risk of developing depression compared to non-AIDS orphans.^19,20^ In a cross-sectional survey conducted in Rwanda, it was found that having both parents deceased increased the risk of developing depression by 25.07 times compared to adolescents having both parents alive.^18^ This study found that a lack of social support increased the chances of developing depressive symptoms in ALWHIV.^18^ In a study by Lwidiko et al., childhood deprivation, having an unemployed caregiver and residing in a rural area predicted the presence of depressive symptoms.^17^ The HIV-related clinical factors include poor treatment adherence, low CD4 count at antiretroviral therapy (ART) initiation, unsuppressed viral load, opportunistic infections and poor tolerance to ART.^13^ In a cross-sectional study conducted in Ethiopia, ALWHIV with poor adherence to ART were found to be 1.73 more likely to develop depressive symptoms.^13^ In this study participants who had a history of opportunistic infections were also found to be at a higher risk of developing depressive symptoms.^13^ Recognising depressive symptoms among adolescents living with HIV (ALWHIV)/AIDS is vital because depression is associated with poor adherence to ART, risky sexual practices and suicidal behaviours.^8^ The integration of mental health services and psychosocial support with HIV management programmes as recommended by the World Health Organization (WHO) and the UNAIDS (The Joint United Nations Programme on HIV/AIDS) results in improved outcomes in common mental disorders.^11,21^ The South African national ART guidelines also recommend routine mental health screening as part of adherence support.^22^

Despite these findings and the recommendations by the WHO, UNAIDS and the South African National ART Guidelines, the screening for mental health problems among ALWHIV is not routinely integrated as part of HIV treatment programmes in the West Rand Health District in Johannesburg. In addition, research on mental health issues of children and adolescents lags considerably behind that of adults.^23^ This is found particularly in resource-limited settings such as South Africa.^5^ Further research is required into the prevalence of mental illness in the specific population of ALWHIV especially now that HIV programmes have been integrated into PHC in the West Rand Health District. While one of the district hospitals, Yusuf Dadoo Hospital, has a designated HIV clinic, it does not have a dedicated HIV clinic with a multidisciplinary team for children and adolescents. The high prevalence of depressive symptoms among ALWHIV demands a more holistic approach and the integration of mental health services into HIV management programmes. Preventative strategies, including psychosocial interventions and early identification of high-risk populations, are also vital.

This study aimed to establish the occurrence of depressive symptoms among ALWHIV attending general primary health clinics and one antiretroviral (ARV) clinic in the West Rand Health District in Johannesburg. The researchers hypothesised that HIV-positive adolescents attending general primary health clinics are at high risk of developing depressive symptoms. The objectives of the study were to determine the prevalence of depressive symptoms in ALWHIV attending the general primary health clinics in the West Rand using the Patient Health Questionnaire for Adolescents (PHQ-A). Furthermore, the sociodemographic profile and HIV-related data were described.

## Research methods and design

### Study design, setting and sampling techniques

A cross-sectional study of ALWHIV attending the Phedisong HIV clinic at Dr Yusuf Dadoo Hospital, a 245-bed facility, and five PHC clinic facilities in the West Rand Health District, Johannesburg, was conducted from January to November 2022. The district has 51 PHC facilities and two district hospitals, which serve the urban and peri-urban areas of the district. There are clinics that were excluded from the study as they did not have any ALWHIV. Patients from the Phedisong clinic are transferred to PHC facilities once they are stable. These PHC clinics provide HIV/AIDS and tuberculosis (TB) related treatment using the integrated care model for these illnesses. Currently, ALWHIV is only referred to the PHC medical officer in that particular clinic or to the specialized community psychiatry clinics if they present with psychiatric symptoms during their routine HIV management appointment.

A convenience sampling method was employed to recruit the participants. Participants included HIV-positive male and female adolescents from the age of 10 to 19 years who were on ARVs. The participants could read and write English and knew their HIV status prior to recruitment and enrolment. Adolescents who were unable to consent or who did not have a guardian to give consent were excluded.

The potential participants and their parents or caregivers were contacted by the clinic staff to obtain permission for the researcher to access the personal information contained in their files. The investigator then obtained the list of ALWHIV who gave permission from each facility. The adolescents together with their parents or caregivers were recruited telephonically to participate in the study and a research enrolment day was scheduled by the researcher. The enrolment day was scheduled to coincide with the participants’ scheduled HIV clinic appointment date where possible. The investigator confirmed the disclosure of HIV status using the clinic file and also from the parent or caregiver before recruitment. On the scheduled enrolment and participation day, information about the study was shared again and informed written consent and assent were obtained from the participant’s parent or caregiver.

### Sample size

Sample size estimation was conducted using the Stata 18 Statistical program.^24^ The study used an earlier prevalence of 31.4% [32/102] depressive symptoms in ALWHIV reported by Mukangabire et al., (2021) among adolescents to estimate the current sample size.^18^ There was no specific formula used to calculate the sample size per facility as all ALWHIV in each facility were included in the study. The 95% confidence interval (CI) with an alpha of 5% and an error margin of 12% was applied to estimate the magnitude of depressive symptoms among ALWHIV in the West Rand health district. The 12% error margin was used because this was the first time a study of this nature was conducted in this district. This resulted in a sample size of 123 that was considered statistically significant.

### Data collection and measurement

Data were obtained using a structured socio-demographic questionnaire, which was completed by the participants. The HIV-related clinical data were obtained from the participants’ files. Screening for depressive symptoms was carried out using the PHQ-A, which is a modified version of the PHQ-9. The PHQ-A is a validated instrument for screening, diagnosing, monitoring and measuring the severity of depressive symptoms in adolescents.^25^ This tool is modified specifically for children and adolescents and it consists of 11 questions that are consistent with the DSM-5 diagnostic criteria for a major depressive episode and an additional two items that are for screening for suicidal behaviours.^25^ Scores on the PHQ-A range from 0 to 27 and scores between 0 and 4 indicate no depressive symptoms, 5 to 14 mild to moderate depressive symptoms, 15 to 19 moderate to severe depressive symptoms and 20 or more indicate severe depressive symptoms.^25^ In this study, participants with PHQ-A scores ≥ 5 were classified as having depressive symptoms.

### Data analysis

The data for this study were collated on Microsoft Excel™ and statistically analysed using the Stata 18 statistical program.^24^ The prevalence of depressive symptoms was calculated as a percentage of the total sample size with 95% CI. The prevalence of depressive symptoms was estimated as having depressive symptoms in the 2 weeks prior to the administration of the PHQ-A, and in the past year. The data set comprised mainly of categorical data and some continuous data, which were further recorded into frequencies. Categorical variables were analysed using Pearson’s Chi-squared test of independence and validated for strength of association using Cramer’s values (Cramer’s V), with a value of close to zero indicating a lack of or weak association and close to one (value > 0.25) indicating a very strong association.^26,27^ The descriptive statistics are presented as tables.

Variables associated with depressive symptoms were determined based on a significance level of *p* ≤ 0.05, with *p* > 0.05 indicating a non-significant association. With regard to bivariate analysis, odds ratios (OR), 95% CI, and *p*-values were calculated to assess the risk factors for depressive symptoms. Variables with significance levels of 20% or below in the bivariate analysis were included in the multivariate analysis. Overfitting was estimated using the ‘least likely’ command to check for extreme outliers. Interactions between variables were estimated and model selection was based on Akaike’s and Schwarz’s Bayesian information criteria to identify the best-fitting model.^28^

### Definitions in the study

In the absence of definitive clinic notes ascribing mode of infection (MOI), the MOI was measured using a cut-off age of 10 years based on existing sub-Saharan African paediatric HIV cohorts.^26^ Those who began ARV drugs before the age of 10 were designated as perinatally infected and those who began ARVs at 10 years of age and older were designated as behaviourally infected.^29^

### Ethical considerations

Ethical clearance to conduct this study was obtained from the University of the Witwatersrand, Faculty of Health Sciences Human Research Ethics Committee (Medical), (No. M210659). Research site approval was obtained from the West Rand District Council’s Research Committee. Permission to share the patient’s personal details with the research investigator for recruitment purposes was telephonically obtained from caregivers or directly from the adolescents 18 years and older by the allocated clinic nurse. Written informed assents and consents were obtained prior to data collection from the participants and caregivers for participants younger than 18 years by the investigator. To protect the participant’s identity, a unique number was then assigned to the participant’s file. Hard copies of the data were stored in a lockable cabinet in the principal investigator’s office and the electronic spreadsheet was password-protected. Only the principal investigator, supervisors and statistician had access to the data.

The investigator reviewed the PHQ-A immediately after completion and participants reporting psychological distress and in need of immediate mental health care were referred urgently for risk assessment and containment on the same day. Those with moderate to severe depressive symptoms or severe depressive symptoms were referred to the nearest specialised community psychiatric services.

## Results

### Sociodemographic characteristics

Overall, 158 adolescents were recruited to participate in the study and 125 were enrolled and screened. Thirty-three adolescents were excluded from the study for several reasons including being unaware of their HIV status (*n* = 24), school commitments (*n* = 8) and no reasons given (*n* = 1). There were almost equal female (*n* = 64, 51.2%) and male (*n* = 61, 48.8%) participants. The mean age of participants was 16.1 years, standard deviation (s.d.) ± 2.4 (Table 1). Eighty-four (67.2%) participants were in high school and the majority attended mainstream schooling (*n* = 108, 86.4%) (Table 1). Significantly more adolescents repeated grades at school (*n* = 77, 61.6%). In terms of caregiver support, the majority of participants (87, 69.6%) had either one (*n* = 46, 36.8%) or both parents (*n* = 41, 32.8%) as caregivers. The majority of the caregivers were employed (*n* = 68, 54.4%) (Table 1).

**TABLE 1:** The sociodemographic characteristics of adolescents living with HIV in the West Rand District, Johannesburg (*N* = 125).

Variables	Total	
	Participants	%
**Sociodemographic characteristics**		
**Age of adolescents (mean age = 16.1 years, s.d. ± 2.4)**		
10–14 years	34	27.2
15–19 years	91	72.8
**Gender**		
Female	64	51.2
Male	61	48.8
**Level of education**		
Primary school education	29	23.2
High school education	84	67.2
Tertiary education	12	9.6
**Type of school**		
Mainstream	108	86.4
Special	11	8.8
Remedial	6	4.8
**Repeated a grade**		
No	48	38.4
Yes	77	61.6
**Caregiver**		
Both parents	41	32.8
Father only	6	4.8
Mother only	40	32.0
Others (such as grandparents, siblings, aunts, uncles, etc.)	38	30.4
**Deceased parent**		
Both parents	15	12.0
Father	15	12.0
Mother	22	17.6
None	73	58.4
**Parent or caregiver’s occupation**		
Formally employed	68	54.4
Self-employed	15	12.0
Unemployed	42	33.6

### Psychiatric and HIV-related characteristics

A notable 9.6% of ALWHIV reported a previous diagnosis of major depressive disorder (Table 2). However, the majority of the participants did not report a previous history of other psychiatric diagnosis (*n* = 118, 94.4%) or substance use history (*n* = 110, 88%) (Table 2). Only 12% of participants reported substance use, with cannabis, alcohol and nicotine being the only substances that were reported by the participants (Table 2). The study did not further test the validity of the participants’ subjective responses regarding substance use. The majority of the participants demonstrated vertical transmission (*n* = 106, 84.8%) and early ART initiation at ≤ 10 years of age (*n* = 86, 68.8%). Eighty-one per cent of participants (*n* = 101) were adherent to ART. Most of the participants did not have other chronic medical illnesses (*n* = 120, 96.8%) and HIV-related opportunistic infections (*n* = 85, 68.5%). A high number of participants had a CD4 count of less than 500 cells/mm^3^ (*n* = 64, 55.2%) at initiation. Many of the participants (*n* = 88, 72.7%) had full viral suppression (VL < 50 copies/ML).^22^

**TABLE 2:** Psychiatric characteristics of adolescents living with HIV in the West Rand District, Johannesburg (*N* = 125).

Variables	Total	
	Participants	%
**Psychiatric characteristics of ALWHIV**		
**History of substance use**		
No	110	88.0
Yes	15	12.0
**Type of substance**		
Alcohol	2	13.3
Cannabis	11	73.3
Other (nicotine)	2	13.3
**Past history of depression**		
Yes	12	9.6
No	108	86.4
Unknown	5	4.0
**History of other mental illness**		
Yes	7	5.6
No	118	94.4

ALWHIV, adolescents living with HIV.

### Prevalence of depressive symptoms

The overall prevalence of depressive symptoms was 44.8% (*n* = 56) on PHQ-A (Table 3). These participants had experienced depressive symptoms in the past 2 weeks prior to the PHQ-A being administered. In this study sample, 40.8% (*n* = 51) of participants reported depressive symptoms in the past year (Table 3). In terms of the severity of the depressive symptoms, 36.8% (*n* = 46) reported mild to moderate depressive symptoms, 6.4% (*n* = 8) moderate to severe symptoms and 1.6% (*n* = 2) severe depressive symptoms (Table 3).

**TABLE 3:** Depressive symptoms on Patient Health Questionnaire for Adolescents (PHQ-A) of adolescents living with HIV in the West Rand District, Johannesburg (*N* = 125).

Variables	Total	
	Participants	%
**Depressive symptoms on PHQ-A**		
**Depressive symptoms in the past 2 weeks**		
Mild to moderate	46	36.8
Moderate to severe	8	6.4
Severe	2	1.6
None	69	55.2
**Depressive symptoms in the past year**		
No	74	59.2
Yes	51	40.8
**Suicide ideation**		
No	104	83.2
Yes	21	16.8
**Previous suicide attempt**		
No	100	80.0
Yes	25	20.0
**Difficulties at school or home or getting along with others**		
Not difficult	90	72.0
Yes difficult	35	28.0

PHQ-A, Patient Health Questionnaire for Adolescents (PHQ-A).

The prevalence of depressive symptoms was higher among the female participants (58.9%, *n* = 33) (Table 4). Concerning the participant’s age, the prevalence was higher for older adolescents (15–19 years) (87.5%, *n* = 49) than younger adolescents (10–14 years) (12.5%, *n* = 7) (Table 4). The majority of the participants with depressive symptoms had suppressed VL (*n* = 35, 66%) and low CD4 counts at initiation (*n* = 30, 57.7%) (Table 4). This prevalence of depressive symptoms was the same in participants with deceased parent or parents and in those with both parents alive (Table 4). The prevalence of depressive symptoms was strongly associated with older adolescents, previous suicidal attempts and difficulties at school or home or getting along with others (Table 4).

**TABLE 4:** Prevalence of depressive symptoms in adolescents living with HIV and associated factors in the West Rand District, Johannesburg.

Characteristics	Depressive symptoms in the past 2 weeks							
	Yes (*N* = 56)		No (*N* = 69)		Total		*p*	Cramer’s *V*
	*N*	%	*N*	%	*N*	%		
**Gender**							0.041	0.1825
Female	33	58.9	28	40.6	61	48.8	-	-
Male	23	41.1	41	59.4	64	51.2	-	-
Total	56	100.0	69	100.0	125	100.0	-	-
**Age (years)**							0.001	0.2976
10–14	7	12.5	27	39.1	34	27.2	-	-
15–19	49	87.5	42	60.9	91	72.8	-	-
Total	56	100.0	69	100.0	125	100.0	-	-
**Deceased parent**							0.086	0.1535
Yes	28	50.0	24	34.8	52	41.6	-	-
No	28	50.0	45	65.2	73	58.4	-	-
Total	56	100.0	69	100.0	125	100.0	-	-
**VL (Copies/ML) latest**							0.145	0.1326
VL < 50	35	66.0	53	77.9	88	72.7	-	-
VL > 50	18	34.0	15	22.1	33	27.3	-	-
Total	53	100.0	68	100.0	121	100.0	-	-
**CD4 count (cells/mm^3^) at initiation**							0.075	0.0457
CD4 < 500	30	57.7	34	53.1	64	55.2	-	-
CD4 > 500	22	42.3	30	46.9	52	44.8	-	-
Total	52	100.0	64	100.0	116	100.0	-	-
**Suicide ideation**							0.027	0.1976
Yes	14	25.0	7	10.1	21	16.8	-	-
No	42	75.0	62	89.9	104	83.2	-	-
Total	56	100.0	69	100.0	125	100.0	-	-
**Previous suicide attempt**							0.001	0.4344
Yes	22	39.3	3	4.3	25	20.0	-	-
No	34	60.7	66	95.7	100	80.0	-	-
Total	56	100.0	69	100.0	125	100.0	-	-
**Suicidal behaviours**							0.001	0.3931
Yes	25	44.6	7	10.1	32	25.6	-	-
No	31	55.4	62	89.9	93	74.4	-	-
Total	56	100.0	69	100.0	125	100.0	-	-
**Difficulties at school or home or getting along with others**							0.001	0.3698
Yes	26	46.4	9	13.0	35	28.0	-	-
No	30	53.6	60	87.0	90	72.0	-	-
Total	56	100.0	69	100.0	125	100.0	-	-
**Referred for intervention**							0.001	-
Yes	38	67.9	2	2.9	40	32.0	-	-
No	18	32.1	67	97.1	85	68.0	-	-
Total	56	100.0	69	100.0	125	100.0	-	-

Note: CD4 < 500 cells/mm^3^ = low CD4 count; CD4 > 500 cells/mm^3^ = high CD4 count; VL = viral load; VL < 50 copies/ML = fully suppressed; VL > 50 copies/ML = not fully suppressed.^22^

Overall, 25.6% (*n* = 46) of participants reported suicidal behaviours on the PHQ-A with 16.8% (*n* = 21) suicidal ideation and 20% (*n* = 25) reporting a previous suicide attempt (Table 4). Twenty-eight per cent (*n* = 35) of adolescents reported difficulties at school or home or getting along with others (Table 4), and only two of these participants were already attending psychotherapy. After the completion and assessment of the PHQ-A, 32% (*n* = 40) of participants were referred for further assessment and management (Table 4) and three participants were referred for urgent intervention.

### Factors associated with the prevalence of depressive symptoms

Multivariate analysis showed that ALWHIV with difficulties at school or home environment or getting along with others and those who reported a previous suicide attempt were 3.6 (aOR 3.59, 95% CI:1.17–11.03) and 6.9 (aOR 6.93, 95% CI:1.39–34.55) times likely to develop depressive symptoms, respectively, compared to those who did not have any difficulties and those who never attempted suicide before (Table 5).

**TABLE 5:** Logistic regression of independent factors of depressive symptoms in adolescents living with HIV in the West Rand health district.

Risk factors	Depressive symptoms					
	cOR	95%CI	*p*	aOR	95%CI	*p*
**Age (years)**						
10–14	Ref.	-	-	Ref.	-	-
15–19	4.5	1.78–11.38	0.001	2.41	0.80–7.25	0.117
**Gender**						
Male	Ref.	-	-	Ref.	-	-
Female	2.1	1.03–4.30	0.042	2.11	0.81–5.49	0.125
**Difficulties at school or home or getting along with others**						
No	Ref.	-	-	Ref.	-	-
Yes	5.78	2.42–13.89	< 0.001	3.59	1.17–11.03	0.026
**Previous suicidal attempt**						
No	Ref.	-	-	Ref.	-	-
Yes	14.23	3.98–50.96	< 0.001	6.93	1.39–34.55	0.018

cOR; crude odds ratio; aOR, adjusted odds ratio; Cl, confidence interval.

## Discussion

Similar to other studies, the prevalence of depressive symptoms among the ALWHIV in this study was high (44.8%). This prevalence was higher among female adolescents (58.9%) and in the older age group of 15–19 years (87.5%). Evidence shows that the prevalence of depressive symptoms is higher among adolescents with HIV/AIDS when compared to adolescents without HIV/AIDS in the general population.^30^ In a cross-sectional study by Ekat et al., using a cutoff score of ≥ 9 on the PHQ-9, the prevalence of depressive symptoms was found to be high (39%).^31^ Gaitho et al., using the Home Environment, Education and Employment, Activity, Sexuality, Suicide and depression traits (HEADSS) tool combined with the PHQ-9 in their study, found a 53% prevalence of depressive symptoms in 270 ALWHIV, which was higher than our findings.^12^ Similar to our study, this prevalence was also higher among the older age group of 15 to 19 years old (64.8%).^12^ In a study by Bankole et al., using the Mini International Neuropsychiatric Interview, which is a diagnostic tool for children and adolescents, the prevalence of depression was 41.9%.^32^ Although this study used a diagnostic tool, the prevalence of depression was similar to our prevalence using a screening tool. Because our study was conducted in the third year of the coronavirus disease 2019 (COVID-19) pandemic, consideration could be made that the psychosocial impact of the COVID-19 pandemic could have further contributed to this high prevalence of depressive symptoms. However, there have been studies that were conducted prior to the COVID-19 pandemic that have reported similar prevalence rates of depression among ALWHIV.^33^

While our study found a high prevalence of depressive symptoms in ALWHIV, there are, however, studies that have reported lower prevalence rates.^1,7,9,10,13,17,18,32,34,35,36^ In a study by Buckley et al., HIV-positive and HIV-negative adolescents were screened for depressive symptoms using the PHQ-A and the prevalence of depression was similar in the two groups of adolescents with an overall prevalence of 14%, which was lower than that of our study.^36^ The variation in the prevalence of depression found in these studies could be related to the different socio-demographic characteristics of the participants, the study sites, and the screening and diagnostic tools used.^1,7,9,10,13,17,18,32,34,35^ Additional factors reported in previous literature include biological factors such as the clinical stage of the HIV illness, the presence of opportunistic infections and adherence to ART. Despite the high prevalence of depressive symptoms in our study, most of the adolescents experienced mild-moderate symptoms, which is comparable to previous studies.^18,34^ The implication of this finding is that there is an opportunity for early identification and intervention in these adolescents before they develop severe symptoms of depression. A notable 2.4% of adolescents required urgent psychosocial intervention. These interventions including psychological, behavioural and social have been identified to be valuable when delivered to general adolescent populations to lessen the risk of poor mental health outcomes and enhance psychosocial well-being.^37^ In addition to the high prevalence of current depressive symptoms, 9.6% of the participants were previously diagnosed with a major depressive disorder. This meant that this population was already vulnerable to developing further mental illness. This further highlights the need for an integrated service delivery model for HIV care. In previous literature, the overall high prevalence of depression in ALWHIV is thought to be related to the vulnerable stage of cognitive and socio-emotional development in this age group with a particular sensitivity to emotions and stress.^12,38^ In addition, HIV interrupts critical developmental processes resulting in ALWHIV susceptible to a wide range of mental, physical and psychological adverse effects.^12^

In this study, the reported suicidal behaviour by the adolescents on the PHQ-A was 25.6% of which 16.8% reported suicidal ideation in the past month and 20% reported previous suicide attempts. This finding was similar to the general population but lower than that reported among ALWHIV in other studies.^30,36,39^ In the study by Buckley et al., the overall rate of suicide ideation was 35%, which was higher among HIV-positive adolescents (42%) compared to HIV-negative adolescents (28%).^36^ Additionally, our study revealed that a history of suicide attempts was linked to an increased likelihood of experiencing depressive symptoms.

In previous studies, a high prevalence of depressive symptoms was reported to be linked to non-adherence, low CD4 count at initiation, high viral load and presence of opportunistic infections.^8,12,13,31,34,35,40^ Non-adherence could result in a high viral load, immunosuppression and an increase in opportunistic infections that might contribute to the onset of depressive symptoms.^34^ Because of the improvement in HIV management, with early initiation of children on ART and the presence of parental or caregiver support ensuring good adherence to ART, there has been a decline in opportunistic infections.^22,41^ This study found most adolescents were adherent to ART, had achieved full viral suppression and had no opportunistic infections. This is consistent with good clinical response seen in early ART initiation. Despite good clinical ART response observed in this study, there was a high prevalence of depressive symptoms. It may be argued that this may be linked to the finding that some of these adolescents are having difficulties at school, in the home environment or getting along with others. However, the CD4 count at initiation was low in the majority of the adolescents with depressive symptoms which was consistent with previous studies.^8,12,13,31,34,35,40^

A notable finding in this study is that only 12% of the participants reported using substances, which is much lower than the prevalence of 41.6% that was found in a previous review and meta-analysis in sub-Saharan Africa.^42^ This could be related to the fact that the adolescents were accompanied by their parents or caregivers which could impact self-reports. In this study, there was a high number of adolescents who had either one or both parents deceased (41.6%). Having one or both parents deceased has been shown to increase the likelihood of developing depression.^18^ However, in this study there was no significant difference in the prevalence of depressive symptoms among adolescents with deceased parents when compared to those who have both parents.

Concerning academic performance and psychosocial difficulties, 61.6% and 28% of the participants experienced difficulties, respectively, which is consistent with available literature.^43^ In a systematic review by Opstal et al., children and ALWHIV in high-income countries were found to experience more difficulties in various areas of school performance compared to HIV-negative adolescents.^43^ Furthermore, we found that having difficulties in school or at home, along with challenges in social relationships, corresponded with an elevated risk of depressive symptoms. In a study by Rukuni et al., significant impairments in the memory and learning domains were reported on the Washington Group/UNICEF Child Functioning and Disability Module in ALWHIV.^44^ These challenges included slowed academic achievement, difficulties making friends and less capacity for engagement in classes and activities.^44^ In addition, repeating a grade has been linked with cognitive deficits from childhood HIV infection, school absenteeism, depression and other psychosocial stressors.^7,12,32,44,45^

This study further contributes to knowledge about mental health screening in ALWHIV. There were however several limitations to this study. The study’s outcomes, while informative, should be interpreted with a degree of caution because of the small sample size. Because this study was a cross-sectional study, we could not infer causal relationships. Certain file-based data were missing from the retrospective analysis. Because the participants were accompanied by their caregivers, social-desirability bias may have influenced how they responded to some questions. There were also challenges with data collection as adolescents were booked on any day for their clinic visits with no structured adolescent clinic for this high-risk group. Only one clinic in the district had a dedicated structured adolescent clinic with support groups. There was no control group, therefore, we could not determine if the depressive symptoms are specific to HIV-infected adolescents or a characteristic of adolescents dealing with any chronic illness. The findings of this study could not be generalised as the study was conducted in one district in Johannesburg.

## Conclusion

Our study shows a high prevalence of depressive symptoms in ALWHIV. Our findings showed that psychosocial difficulties and previous suicide attempts were predictive factors of depressive symptoms. These findings emphasise the need for integrating mental healthcare into routine HIV/AIDS, TB and non-communicable disease (NCD) programmes as recommended by WHO and the South Africa national ART guidelines.^21,22^ This integration will address the high unmet mental health needs for ALWHIV in the primary healthcare setting by facilitating entry onto onsite mental health screening and psychosocial support services for further assessment and referral to specialised psychiatric care if needed. The integration should also equip healthcare providers to develop a stigma-free and adolescent-friendly service that also supports parents and caregivers in timeous disclosure of the HIV status to their children. Further research is needed to determine the causal relationship between the variables identified as depression predictors.
